# Head-to-Head Comparison of FAP Antibody Clones for Tissue Expression Analysis

**DOI:** 10.3390/antib15050093

**Published:** 2026-10-08

**Authors:** Elena Shmakova, Anna Kalinchuk, Elisaveta Tsarenkova, Pavel Bazhenov, Vlada Vasilchuk, Dmitry Loos, Liubov Tashireva

**Affiliations:** 1 Laboratory of Molecular Therapy of Cancer, Cancer Research Institute, Tomsk National Research Medical Center, Russian Academy of Sciences, Tomsk 634050, Russiapavel.nr@yandex.ru (P.B.);; 2 The Department of General and Molecular Pathology, Cancer Research Institute, Tomsk National Research Medical Center, Russian Academy of Sciences, Tomsk 634050, Russia; loos.d@yandex.ru

**Keywords:** fibroblast activation protein (FAP), immunohistochemistry (IHC), antibody validation, cancer-associated fibroblasts (CAFs), breast cancer, colorectal cancer, lung cancer, head and neck cancer, tumor microenvironment, stromal staining, tumor cell staining

## Abstract

**Background:** Fibroblast activation protein (FAP) is a promising target for cancer diagnosis and therapy, but the specificity and sensitivity of anti-FAP antibodies used for immunohistochemistry (IHC) vary considerably. A head-to-head comparison of commercially available clones is needed to guide antibody selection, thereby standardizing FAP IHC protocols and ensuring reliable patient stratification for FAP-directed therapies. **Methods:** We evaluated three anti-FAP clones—FAP/4851 (mouse monoclonal), JA56-11 (recombinant rabbit), and EPR20021 (rabbit monoclonal)—in formalin-fixed, paraffin-embedded tissues from 137 patients with breast (*n* = 29), colorectal (*n* = 27), lung (*n* = 42), and head and neck squamous cell carcinoma (*n* = 39). FAP expression was assessed by manual semiquantitative scoring (H-score and percentage of positive cells) and, for a validation subset, by QuPath-based digital image analysis. The study included negative (normal liver/skeletal muscle) and positive (WI-38 cell block) controls. **Results:** Clones JA56-11 and EPR20021 showed specific stromal staining, whereas FAP/4851 exhibited intense non-specific reactivity and was excluded from quantitative analysis. EPR20021 demonstrated significantly higher H-scores than JA56-11 in all four cancer types (Wilcoxon test, *p* ≤ 0.0179), resulting in a higher overall detection rate under the tested conditions. Aberrant tumor cell expression was detected almost exclusively by EPR20021 in head and neck (59% of cases) and colorectal (59%) cancers. QuPath validation showed good correlation with manual scoring for EPR20021 in the tumor compartment of lung cancer (Spearman’s ρ = 0.701, *p* = 0.024) with small bias (Bland–Altman). **Conclusions:** Under the conditions tested, EPR20021 (Abcam) demonstrated higher staining intensity and detection rates than JA56-11 across several tumor types. Clone FAP/4851 showed nonspecific staining under the conditions used in this study and was therefore not suitable for quantitative analysis in the present experimental setting. Consequently, careful selection of well-validated antibody clones is essential for ensuring reproducible and reliable FAP immunohistochemical analysis.

## 1. Introduction

Fibroblast activation protein (FAP) is a type II transmembrane serine protease that is highly expressed on the surface of cancer-associated fibroblasts (CAFs) in the tumor microenvironment of most epithelial cancers [1,2]. FAP expression is minimal or undetectable in normal adult tissues, making it a promising diagnostic and therapeutic target [2,3]. Beyond its presence on CAFs, emerging evidence indicates that FAP can be aberrantly expressed by malignant epithelial cells themselves, particularly in the context of epithelial-to-mesenchymal transition (EMT) [3].

The prognostic significance of FAP is highly context-dependent, varying with the specific cellular compartment and tumor microenvironment. While elevated stromal FAP expression typically correlates with aggressive tumor behavior and poor clinical outcomes, high FAP levels within specific tumor epithelial compartments or distinct immune-infiltrated niches have paradoxically been associated with prolonged survival in breast, colorectal, lung, and head and neck cancers [4,5,6,7,8]. This positive correlation is frequently mediated by alterations in the immune landscape, where FAP+ fibroblasts have been associated with T cell infiltration. Consequently, elevated FAP expression has been linked to improved outcomes in patients treated with PD-L1 inhibitors, underscoring its potential as a context-dependent biomarker [4,9,10,11].

FAP has emerged as an attractive target for several therapeutic strategies, including FAP-activated prodrugs, radioligand therapy (RLT), and chimeric antigen receptor (CAR)-T cells [12]. More recently, FAP-targeted radioligands (e.g., FAPI-PET) have revolutionized cancer imaging, providing a non-invasive method to assess FAP expression in vivo [13,14]. However, the correlation between FAPI-PET uptake and IHC results depends on the specificity and sensitivity of the antibody used for histopathological validation. This diagnostic accuracy is paramount, as recent studies have highlighted that FAP IHC could serve as a valuable companion screening tool to select and stratify eligible patients for FAP-directed radionuclide and target therapies [4,15,16]. Despite this significant clinical potential, there is a lack of a universally validated diagnostic test for FAP immunohistochemical scoring. However, the clinical value of FAP scoring and selecting an appropriate commercial antibody remains difficult. Many database entries represent the same clone sold under different vendor brands, and many products lack reliable validation for formalin-fixed paraffin-embedded (FFPE) tissues. To address these inconsistencies, a systematic comparison of distinct antibody platforms is required. This study evaluates three different monoclonal antibodies—representing hybridoma-derived rabbit, recombinant rabbit, and mouse IgG platforms—across a multi-cancer cohort to establish a reliable framework for FAP companion diagnostics. To date, no direct comparison has been published for the most common commercial FAP clones, including EPR20021, JA56-11, and FAP/4851. Therefore, a comprehensive head-to-head evaluation of these commercially available antibodies is essential to minimize diagnostic discrepancies, standardize FAP IHC analysis, and establish a reliable framework for comparative microenvironmental profiling.

## 2. Materials and Methods

### 2.1. Patient Cohorts and Tissue Samples

The study included a total of 137 patients with morphologically verified primary breast cancer (*n* = 29), colorectal cancer (*n* = 27), lung cancer (*n* = 42), and head and neck squamous cell carcinoma (*n* = 39). Clinical staging was performed according to the 8th edition of the AJCC/TNM classification for each tumor type [17]. The diagnosis was established based on the examination of surgical resection specimens or diagnostic biopsies according to the respective WHO classification criteria [18]. Detailed clinical and pathological characteristics are available in Table 1).

Formalin-fixed, paraffin-embedded (FFPE) tissue blocks were retrieved from the biobank of the Cancer Research Institute, Tomsk National Research Medical Center. For each case, a representative tumor block containing both malignant epithelial cells and adjacent tumor stroma was selected for whole-section analysis. Serial sections (4 µm thick) were cut for immunohistochemical staining.

The study was approved by the local ethical committee of the Tomsk National Research Medical Center (protocol no. 21 dated 16 December 2025). This work was conducted as part of the registered clinical trial evaluating FAP expression via SPECT/CT imaging (ClinicalTrials.gov ID: NCT07457541, institutional protocol code: ORI-16/2). Written informed consent was obtained from all patients for the use of their residual diagnostic tissue samples for research purposes.

### 2.2. Antibodies and Immunohistochemistry

FFPE tissue blocks from breast, colorectal, lung, and head and neck cancers were sectioned for immunohistochemical staining. Sections were deparaffinized in xylene and rehydrated through a graded ethanol series. Antigen retrieval was performed by heating the sections in EDTA buffer, pH 9.0 at 95 °C for 20 min using a pre-treatment module. Antigen retrieval conditions were optimized during the pilot phase of the study. Heat-induced epitope retrieval for clones EPR20021 and JA56-11 was performed using Tris-EDTA buffer (pH 9.0) for 20 min. For clone FAP/4851, alternative retrieval configurations, including citrate buffer (pH 6.0) and varying incubation intervals, were systematically evaluated; however, Tris-EDTA (pH 9.0) was maintained for consistency as alternative buffers did not reduce non-specific background staining. Endogenous peroxidase activity was blocked with 3% hydrogen peroxide for 5 min. The detailed characteristics of the three anti-FAP antibodies are summarized in Table 2.

The anti-FAP primary antibodies were selected to evaluate three distinct manufacturing platforms validated for FFPE sections. To ensure broad technological representation without technical redundancy, we chose different recombinant rabbit technologies that target distinct regions of the FAP, and a mouse IgG matrix. Clone EPR20021 (Abcam, ab207178) was selected as the primary comparator for the inter-clone analysis, recognizing the glycosylated 100–120 kDa forms of the mature extracellular domain. Clone JA56-11 (Invitrogen, MA5-32670) was used to evaluate an alternative recombinant rabbit IgG platform targeting the specific extracellular domain sequence (aa 1–160). Finally, clone FAP/4851 (Invitrogen, 2191-MSM1-P0) was included as a mouse-derived IgG2c monoclonal option mapping to the N-terminal/transmembrane region (aa 1–200), making it highly suitable for multiplex panels.

Sections were incubated with primary antibodies 25 min at RT in a humidified chamber. The optimal working dilutions for clone EPR20021 (1:300), FAP/4851 (1:1000) clone and clone JA56-11 (1:800) were established by testing 3–4 different concentrations within the manufacturers’ recommended ranges to ensure specific staining and minimize background staining. After washing with PBS, sections were incubated with a polymer-based horseradish peroxidase (HRP)-conjugated secondary antibody (Novolink Polymer Detection System, Leica Biosystems, Newcastle upon Tyne, UK) for 30 min at room temperature. The reaction was visualized using 3,3′-diaminobenzidine (DAB) as the chromogen. Sections were counterstained with hematoxylin, dehydrated, cleared in xylene, and mounted with a permanent mounting medium.

To assess the specificity of the primary antibody staining and to detect potential false-positive signals, a tissue-based negative control was employed. Sections of normal skeletal muscle, tissues known to lack FAP expression, were used as negative controls (Figure S1). The absence of specific immunoreactivity in these control tissues confirmed that the observed staining in the tumor sections was not due to non-specific antibody binding or other technical artifacts. To validate the specificity of the primary antibodies and the entire IHC protocol, a positive control was established using a gelatin-based cell block containing WI-38 human fibroblasts. WI-38 cells are a classical model of FAP-expressing fibroblasts, as the FAP-alpha cDNA was originally cloned from a WI-38 cDNA expression library [19]. This cell line has been extensively validated as a standard positive control for endogenous FAP expression at both the mRNA and protein levels, including verification via Western blot and flow cytometry in benchmark vendor specifications. Strong, specific membranous and cytoplasmic staining of WI-38 cells confirmed the correct performance of the IHC run for each antibody tested. A staining run was considered valid if control tissues showed no specific FAP signal and if the expected staining pattern was observed in this positive control. Non-specific background staining was assessed using an isotype-matched negative control antibody on adjacent sections of the same cell block.

### 2.3. Evaluation and Scoring

Immunohistochemical staining for FAP was evaluated by two independent methods: a quantitative digital image analysis using QuPath software and a manual semiquantitative assessment performed by a board-certified pathologist.

#### 2.3.1. Digital Image Analysis

Digital image analysis was performed using the open-source software QuPath [20] (version 0.7.0). Whole-slide images of FAP-stained sections were acquired using a OneCell Scan 3.0 at ×40 magnification. The annotation (tumor + 100 µm peritumoral stroma) constituted the region of analysis. Normal tissue located further than 100 µm from the tumor edge was excluded from the analysis.

Cell detection was performed using QuPath’s [20] built-in cell detection command with optimized parameters (standard nucleus detection parameters, optical density sum threshold, background radius) and DAB OD mean thresholds of 0.15, 0.27, and 0.47 for classification of weak (1+), moderate (2+), and strong (3+) positivity. After color deconvolution (hematoxylin vs. DAB), cells were classified as FAP-positive based on a predefined DAB intensity threshold. Positive cells were further subdivided into three intensity categories based on the mean DAB optical density in the cell compartment: 1+ (weak), 2+ (moderate), and 3+ (strong). The analysis was run separately for the tumor and peritumoral stromal compartments, which were manually delineated using annotation tools in QuPath.

#### 2.3.2. Manual Semiquantitative Scoring

All stained sections were independently evaluated by a board-certified pathologist. The assessment was performed on the same region of analysis (tumor + 100 µm peritumoral stroma) as defined for digital image analysis. FAP expression was scored separately for the tumor compartment (malignant epithelial cells) and the peritumoral stromal compartment. For each sample, 10 representative fields of view (×200–×400 magnification) were examined. In each compartment, the percentage of cells showing specific membranous or cytoplasmic staining was estimated at three intensity levels: 1+ (weak)—faint brown staining, barely visible at low magnification (×40–×100); 2+ (moderate)—distinct brown staining clearly visible at low magnification; 3+ (strong)—intense dark brown staining with crisp outlines, easily recognizable at low magnification. The H-score was calculated for each compartment using the formula: H-score = [1 × (% 1+ cells) + 2 × (% 2+ cells) + 3 × (% 3+ cells)] giving a theoretical range from 0 to 300. A minimum of 200 cells per compartment were evaluated in each case.

### 2.4. Statistical Analysis

Descriptive statistics are presented as median with interquartile range (IQR). Because the H-score data were not normally distributed (Shapiro–Wilk test, *p* < 0.05), comparisons between EPR20021 and JA56-11 were performed using the Wilcoxon signed-rank test. The Wilcoxon tests for H-score and %-positive were performed independently across the 4 cancer types and 2 compartments. Because each cancer type and compartment represent a distinct biological context, these pairwise tests were evaluated as independent hypotheses without adjustment for multiple comparisons. Correlation between manual and digital H-scores was assessed using Spearman’s rank correlation coefficient. A *p*-value < 0.05 was considered statistically significant. To evaluate the diagnostic concordance and potential interchangeability between clones EPR20021 and JA56-11 within the stromal compartment, percentage scores were dichotomized into negative (≤10%) and positive (>10%) categories based on an exploratory cutoff adapted from established microenvironmental biomarker thresholds [21,22]. The categorical association between the evaluated antibody clones was analyzed using McNemar’s test, and the statistical consistency of the staining results was quantified using Cohen’s kappa coefficient with its 95% confidence interval (CI). All analyses were performed using GraphPad Prism 9.0 (GraphPad Software, USA). 

## 3. Results

### 3.1. FAP Expression Patterns Across Tumor Types

A total of 137 patients were included in this study, comprising breast cancer (*n* = 29), colorectal cancer (*n* = 27), lung cancer (*n* = 42), and head and neck squamous cell carcinoma (*n* = 39). Clinical characteristics are summarized in Table 1.

FAP expression, as determined by immunohistochemistry with two specific antibody clones (JA56-11 and EPR20021), was detected in the tumor microenvironment across all four cancer types, with notable inter- and intra-tumoral heterogeneity. The frequency of FAP-positive cases in the stromal (CAF) compartment varied by tumor type and antibody clone; detailed quantitative data are presented in Table 2. Our findings align with previous reports, which described a high prevalence of FAP-expressing CAFs in colorectal and head and neck tumors, and a more moderate one in breast and lung cancers [23,24,25,26].

FAP was predominantly expressed in CAFs within the peritumoral stroma. Membranous staining of malignant epithelial cells was only occasional, except in head and neck cancers, where a subset of cases showed strong tumor cell positivity (see Section 3.4). In line with previous reports, FAP expression in colorectal cancer was frequently detected at the invasive front and was often heterogeneous within the same tumor [24]. This stromal-predominant pattern was consistent across all four cancer types for clones JA56-11 and EPR20021.

**Figure 1:**
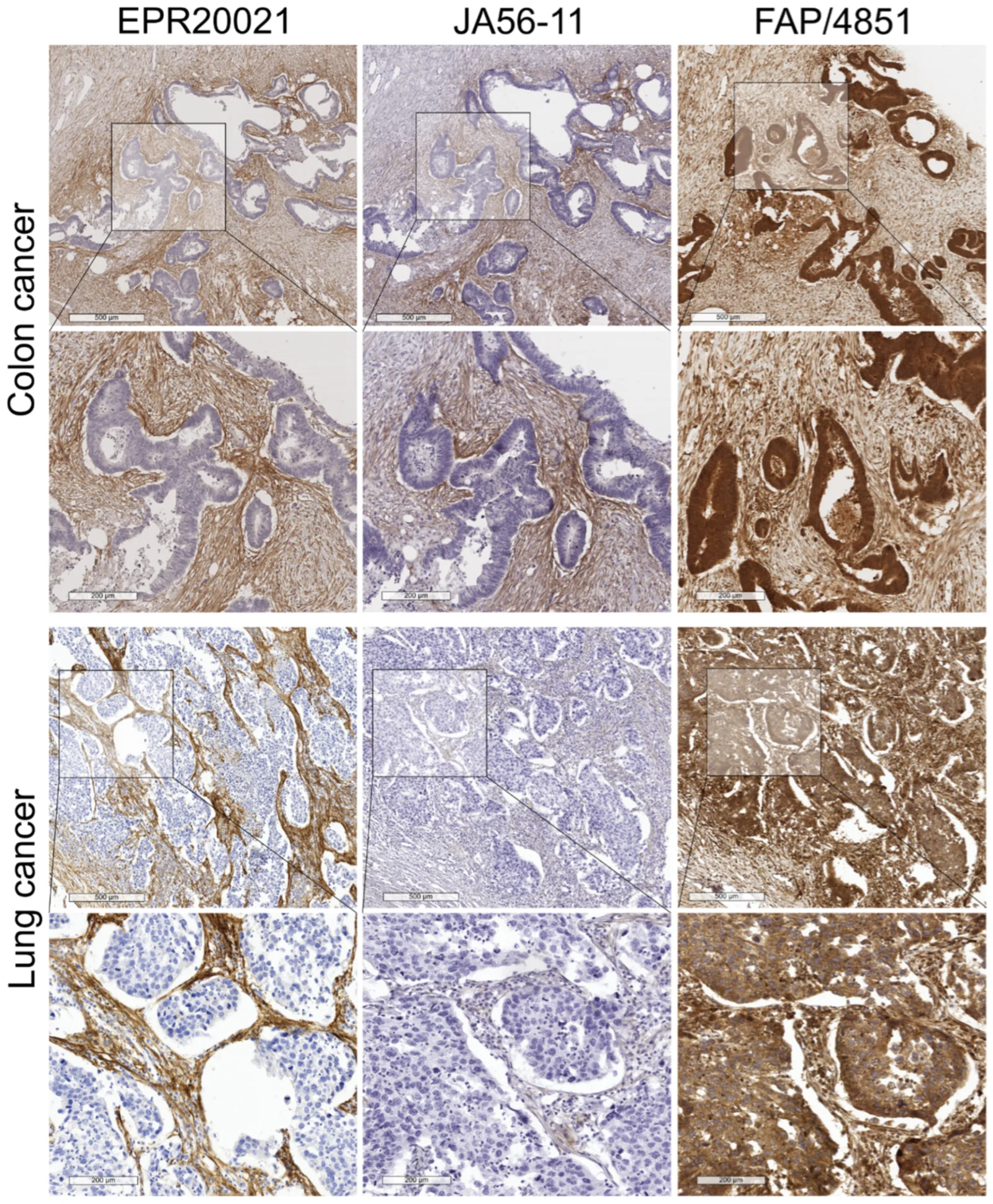

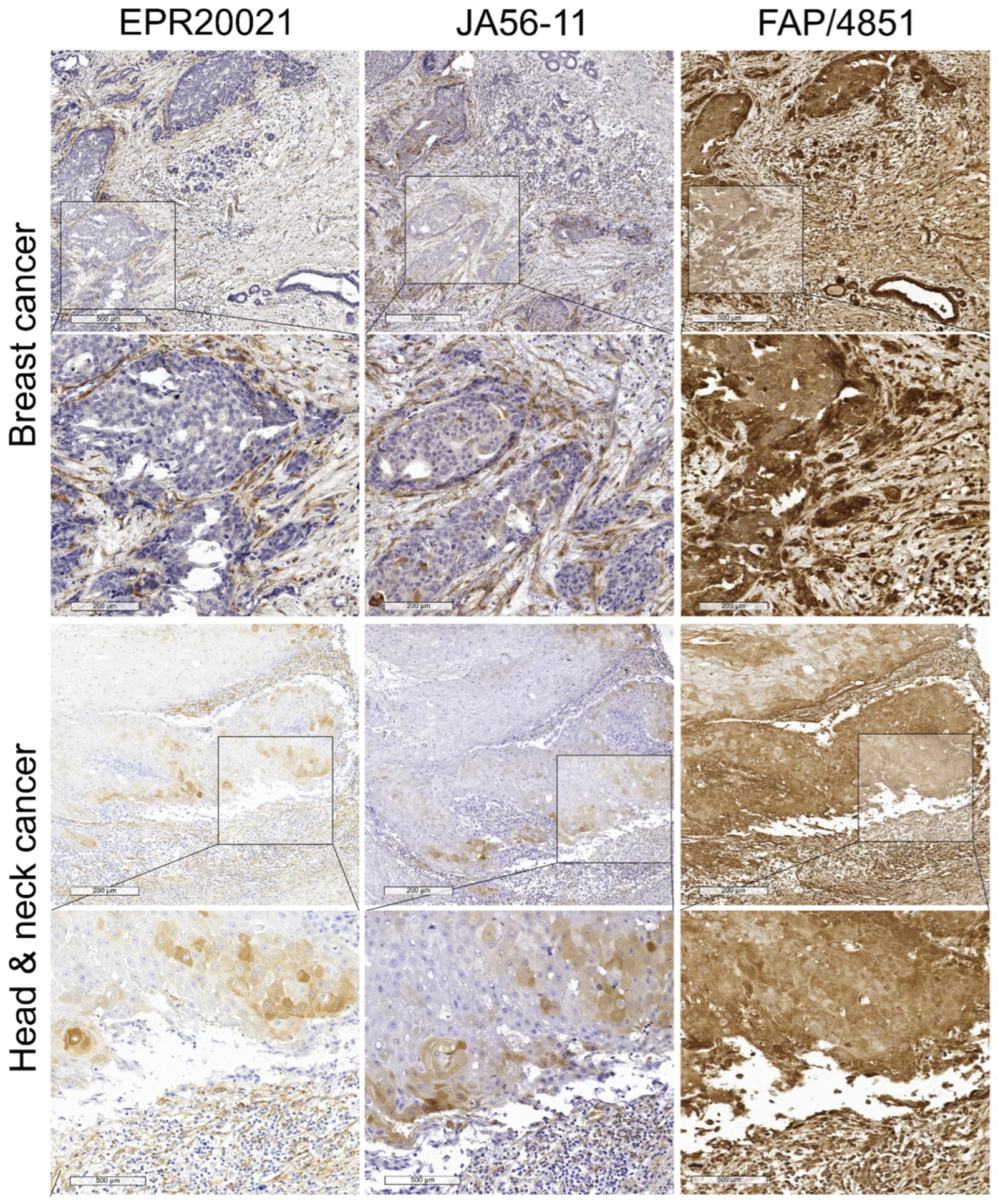
Representative immunohistochemical staining of FAP in breast, colorectal, lung, and head and neck cancers using three anti-FAP antibody clones (EPR20021, JA56-11, and FAP/4851). Rows display representative images for each cancer type, while columns represent the respective antibody clones. Specific positive FAP expression is visualized as brown staining (DAB). Tissue sections were counterstained with hematoxylin (blue). Scale bars for overview and high-magnification images are indicated in the lower-left corners (500 μm and 200 μm, respectively).

Of the three antibodies initially tested, clones JA56-11 and EPR20021 produced specific staining of CAFs and were further evaluated quantitatively. The third clone, FAP/4851, exhibited intense and diffuse non-specific immunoreactivity across all tissue compartments (tumor cells, CAFs, and extracellular matrix) and also stained negative control tissues (normal liver and skeletal muscle). Therefore, this clone was excluded from quantitative comparisons and is presented only qualitatively (Figure 1).

The qualitative differences in staining patterns observed in Figure 1 were further substantiated by manual semiquantitative scoring of all evaluable samples. The results for the stromal compartment are detailed below. Clone EPR20021 consistently produced higher positivity rates and H-scores than JA56-11 across all four cancer types, with the most pronounced differences seen in breast, colorectal and lung cancers.

### 3.2. Manual Semiquantitative Scoring

Manual scoring results for the stromal compartment are summarized in Table 3. Clone EPR20021 showed high positivity rates across all cancer types, with median H-scores ranging from 60 (colorectal) to 145 (lung) and median percentage of positive cells from 50% to 80%. Clone JA56-11 exhibited lower values, particularly in breast and colorectal cancers, where median H-scores were 10 and 10, respectively. Clone 4851 stained nearly all evaluable samples, confirming its non-specific reactivity (Table 3).

To determine whether the observed differences between EPR20021 and JA56 11 were significant, we performed Wilcoxon signed-rank tests for each cancer type. As shown in the integrated quantitative analysis (Table 3), the comparison confirmed that EPR20021 has significantly higher H scores than JA56 11 across all four cancer types: colorectal (*p* = 0.0001), lung (*p* = 0.0002), breast (*p* < 0.0001), and head and neck (*p* = 0.0179). For the percentage of FAP-positive stromal cells, significant differences were similarly observed in colorectal (*p* = 0.0010), lung (*p* = 0.0004), and breast (*p* < 0.0001) cancers, whereas the variation in head and neck cancer did not reach statistical significance (*p* = 0.0539). These results demonstrate that EPR20021 has a higher detection rate for FAP detection across all tumor types, particularly in breast, colorectal and lung cancers. EPR20021 demonstrated a higher detection rate than JA56-11, suggesting its potential suitability for identifying FAP expression across the tested cohort (Figure 1).

Manual scoring of the tumor compartment revealed distinct antibody-specific profiles (Table 4).

Clone EPR20021 detected FAP positivity in a substantial subset of colorectal (59% of cases) and head and neck (59%) cancers, whereas it was only periodically observed in lung (33%) and rarely in breast (3%) tumors. In contrast, clone JA56 11 remained negative in tumor cells across all four cancer types (≤5% positivity).

When comparing the quantitative staining parameters via the paired Wilcoxon signed-rank test, a statistically significant difference between the two antibodies was demonstrated only in colorectal cancer (*p* = 0.0015 for both H score and percentage). Conversely, in lung, breast, and head and neck cancers, the quantitative differences did not reach statistical significance (*p* > 0.05). In the head and neck cancer cohort, this difference was not statistically significant because the staining detected by EPR20021 was weak and focal, as shown by the low median H-score of 5 (IQR 0–25). Consequently, the absolute numerical difference between the two antibodies was too small for the Wilcoxon test to reach significance.

**Figure 2:**
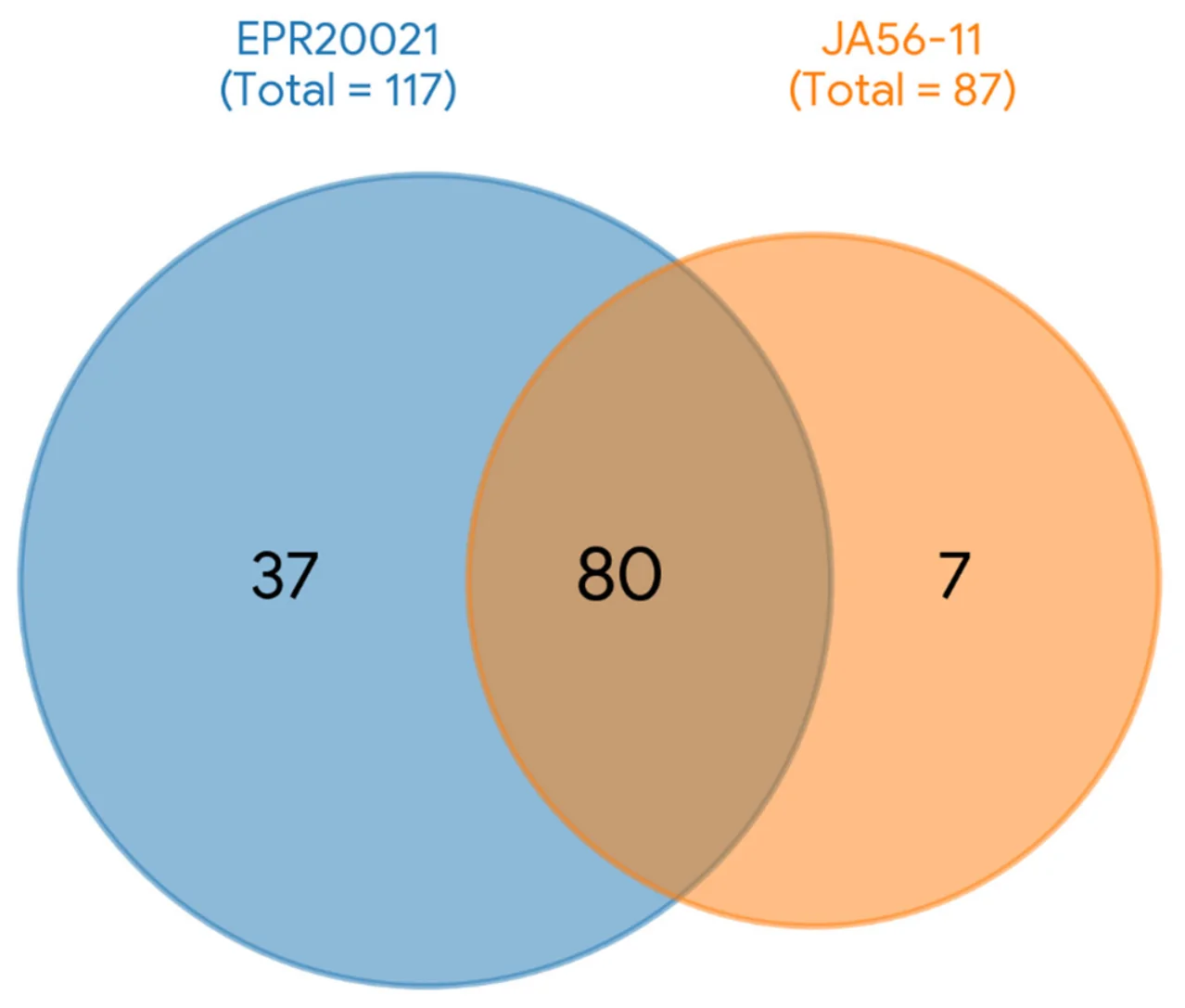
Venn diagram demonstrating the diagnostic intersection and variability of stromal FAP detection between antibody clones. The diagram illustrates the overlap of FAP-positive cases (defined by an exploratory cut-off of >10% vs. ≤10% positive stromal cells) between clone EPR20021 (*n* = 117 total positive cases) and clone JA56-11 (*n* = 87 total positive cases) across the evaluated cohort (*n* = 135 paired samples). The prominent shaded crescent represents the 37 cases (27.4%) that were captured by the EPR20021 clone while remaining negative when evaluated with clone JA56-11.

Based on an exploratory cut-off protocol (defining positivity as >10% vs. ≤10% stromal area) adapted from established immuno-oncology scoring conventions [21,22], categorical classification revealed poor diagnostic equivalence between the antibody clones (Figure 2).

McNemar’s test indicated a systematic difference in the categorical stromal FAP classification between the two antibody clones (*p* < 0.0001, Chi-squared = 19.11). Consistently, Cohen’s kappa coefficient confirmed a low level of agreement between the two methods (κ = 0.173, 95% CI: 0.022–0.324, *p* = 0.015). The overall concordance across 135 paired samples was 67.4% (91/135), with 80 concordant positive and 11 concordant negative results. Crucially, relative to the EPR20021 reference, clone JA56-11 exhibited a lower identification rate, demonstrating undetectable FAP expression in 27.4% of FAP-positive tumors (37/135 cases), whereas only 7 samples (5.2%) were positive exclusively with JA56-11. Overall, FAP expression varies across different tumor types. Compared to the other antibody platforms, clone EPR20021 demonstrates a higher capacity to identify FAP expression with low levels of this marker.

### 3.3. FAP Localization Patterns and Epitope Specificity

**Figure 3:**
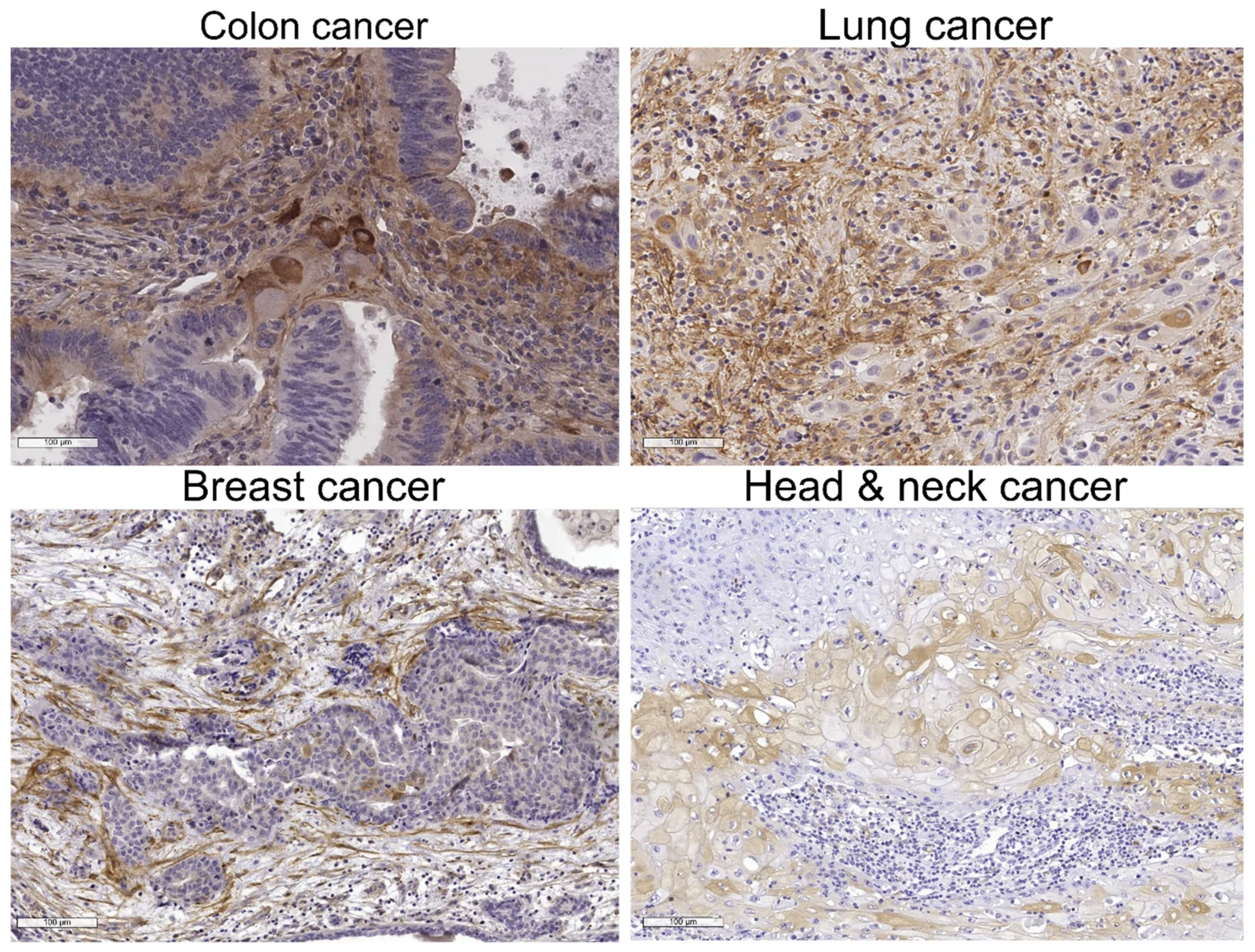
Representative intertumoral immunohistochemical staining of FAP in colorectal, lung, breast, and head and neck cancers using clone EPR20021 anti-FAP antibody.

The subcellular localization of FAP was assessed for two antibody clones, with a focus on the specific clones JA56-11 and EPR20021. Both JA56-11 and EPR20021 clones predominantly stained the membrane of peritumoral CAFs, which is consistent with FAP’s known biology as a type II transmembrane protein [1]. This pattern was consistent across all four cancer types and is considered the specific staining pattern for FAP (Figure 3).

Despite this consistent localization, the FAP expression varied considerably between tumor types. Although FAP was predominantly expressed in the stromal compartment across all tumor types, both the level of expression (as measured by H score) and the frequency of FAP-positive cases varied by cancer type. The highest stromal H scores were observed in lung (median 145, IQR 50–180) and breast (median 100, IQR 50–200) cancers, followed by head and neck (median 80, IQR 50–160) and colorectal (median 60, IQR 10–160) tumors (Table 3). This quantitative pattern is consistent with previous reports showing a high prevalence of FAP-expressing CAFs in colorectal and head and neck cancers (e.g., 79% in colorectal cancer) and a more moderate prevalence in breast and lung cancers (median FAP area coverage of 6.5% and 6.6%, respectively) [24].

Beyond these quantitative differences, we also observed qualitative variations in subcellular distribution. In addition to membranous reactivity, cytoplasmic staining was also observed in a subset of CAFs, particularly in those located at the invasive front. This cytoplasmic staining character may represent an intracellular pooling of FAP or reflect the dynamics of protein synthesis and trafficking.

In addition to the stromal compartment, we assessed FAP expression in the malignant epithelial cell compartment. FAP expression in tumor cells was detected almost exclusively in head and neck and colorectal cancers, where EPR20021 identified positive cases in 23 of 39 (59%) and 16 of 27 (59%) cases, respectively. In contrast, lung and breast cancers showed minimal tumor cell positivity, with EPR20021 detecting FAP in 14 of 42 (33%) lung and 1 of 29 (3%) breast cancers (Table 4). This distinct pattern suggests that FAP expression in malignant epithelial cells is not a common phenomenon but rather is restricted to specific tumor types, possibly reflecting EMT.

### 3.4. Validation of Digital Image Analysis (QuPath)

To assess the reliability of automated quantification, we compared QuPath-derived estimates with manual scoring by an experienced pathologist on a subset of samples representing all four cancer types. The analysis included measurements of the percentage of positive cells and H-scores for the two specific clones (EPR20021 and JA56-11) in both stromal and tumor compartments. Because the data deviated from normality (Shapiro–Wilk test *p* < 0.05 for most comparisons), we used Spearman’s rank correlation, Bland–Altman plots and the Wilcoxon test to evaluate agreement and systematic bias.

**Figure 4:**
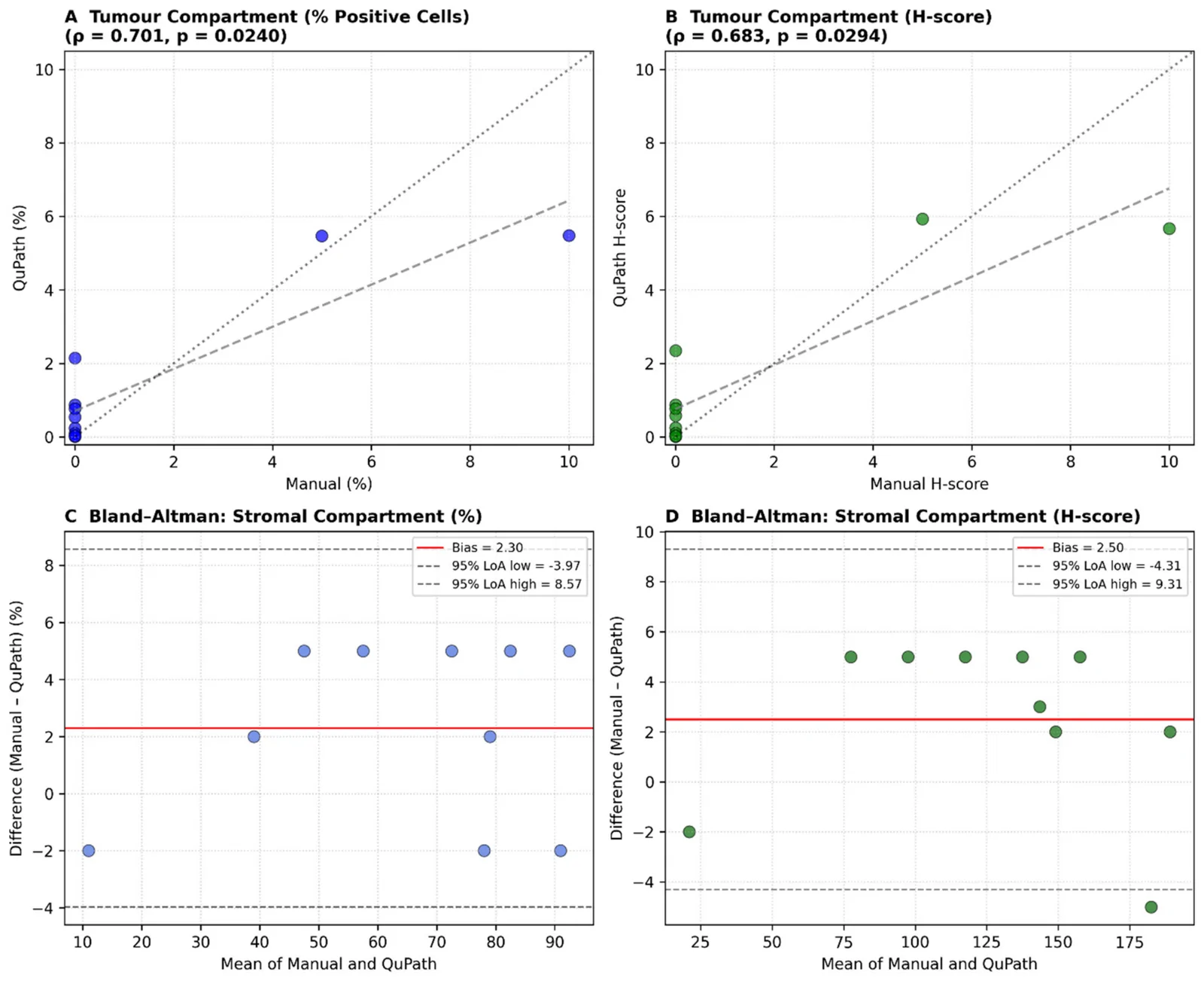
Correlation and agreement analysis between manual pathomorphological scoring and QuPath-based digital image analysis for clone EPR20021 in lung cancer. (**A**,**B**) Scatter plots demonstrating Spearman’s rank correlation (ρ) and corresponding *p*-values for the percentage of positive cells (**A**) and H-scores (**B**) evaluated exclusively within the tumor compartment (*n* = 10). The dashed line represents perfect agreement (y = x), and the dotted line shows the linear regression trend. (**C**,**D**) Bland–Altman plots evaluating systematic bias within the stromal compartment for the percentage of positive cells (**C**) and H-scores (**D**). The solid red line indicates the mean difference (bias), while the dashed lines define the 95% limits of agreement (LoA).

For the tumor compartment, a statistically significant correlation was confirmed between manual and digital quantification for both the percentage of positive cells (ρ = 0.701, *p* = 0.024; Figure 4A) and H-scores (ρ = 0.683, *p* = 0.029; Figure 4B).

However, a notable feature of the tumor compartment profiling was the predominance of zero values across 90% of the validation subset, reflecting the naturally low or restricted epithelial FAP expression in lung cancer. Despite this distribution, the automated cell detection pipeline demonstrated excellent baseline calibration, tightly mirroring manual scores in near-negative settings.

In contrast, the stromal compartment provided a continuous distribution of FAP expression, which was further evaluated via Bland–Altman analysis to detect systematic discrepancies. For the stromal percentage of positive cells, the analysis revealed a small systematic bias of 2.30% (95% limits of agreement [LoA]: –3.97% to 8.57%; Figure 4C), indicating a minor underestimation by the digital algorithm. Similarly, the quantitative H-score evaluation demonstrated a stable systematic bias of 2.50 (95% LoA: –4.31 to 9.31; Figure 4D), with narrow limits of agreement. Taken together, these findings demonstrate a strong quantitative correlation between manual scoring and digital image analysis in the stromal compartment, while highlighting that careful threshold optimization remains mandatory to resolve the complex architectural background of CAF-rich extracellular matrices.

For the clone JA56-11 with a lower detection rate in the lung cancer cohort, the automated quantification pipeline demonstrated variable performance that was highly dependent on the tissue compartment. In the tumor compartment, where antigen density was minimal, the correlation between manual and QuPath-based metrics remained moderate but significant (percentage: ρ = 0.561, *p* = 0.037, *n* = 17; H-score: ρ = 0.561, *p* = 0.037, *n* = 17). However, in the stromal compartment, clone JA56-11 exhibited high correlation parameters (percentage: ρ = 0.799, *p* = 0.0002, *n* = 17; H-score: ρ = 0.820, *p* = 0.0001, *n* = 17). Despite these high correlation coefficients, digital evaluation of JA56-11 in the stroma revealed a substantial systematic underestimation compared to manual scores, driven by the algorithm’s inability to effectively resolve weak, low-intensity staining against background noise. Taken together, these methodological findings indicate that while digital pathology via QuPath offers reproducible, high-throughput advantages for FAP screening, its analytical precision is strictly contingent upon the specific antibody clone and tissue compartment. Automated quantification performs optimally when paired with EPR20021 clone due to its intense signal, whereas clones with weaker reactivity or complex, hypercellular stromal matrices remain prone to algorithmic underestimation, underscoring the continued necessity of manual pathomorphological validation.

A limitation of the manual scoring approach, particularly in the tumor compartment was the predominance of zero values across most cancer types. This predominance of zero values complicates correlation analysis and underscores the need for more sensitive quantification methods. QuPath, despite requiring careful calibration, offers a promising alternative for reproducible, high-throughput FAP assessment, and merits further validation in larger cohorts.

Taken together, the performance of QuPath was highly dependent on the compartment, tumor type and antibody clone. Automated quantification worked reasonably well for EPR20021 in the tumor compartment of lung cancer and demonstrated acceptable diagnostic agreement within the stromal compartment. However, its analytical precision remained limited for the less sensitive clone JA56 11, which was prone to systematic algorithmic underestimation in complex, hypercellular stromal matrices. These discrepancies likely reflect the complex architecture of CAF-rich stroma, variable staining intensity and algorithmic sensitivity to background. Therefore, the primary quantitative results of this study are based on manual scoring, whereas QuPath may be considered a supportive tool for high-throughput screening after further optimization and validation on larger, more homogeneous cohorts [27].

## 4. Discussion

In this head-to-head comparison of three commercial anti-FAP antibodies across four common epithelial cancers, we have shown that EPR20021 (Abcam) yields a significantly higher detection rate than JA56-11 (Invitrogen) for detecting FAP in the stromal compartment, with highly significant differences in all four tumor types (*p* ≤ 0.0179 for H-score). In contrast, clone FAP/4851 showed substantial nonspecific staining under the conditions used in this study and was therefore not suitable for quantitative analysis in the present experimental setting.

The higher staining efficiency of EPR20021 likely reflects differences in epitope recognition or antibody affinity. JA56-11 may recognize a conformation-sensitive epitope that is masked or lost in CAFs with lower FAP expression, whereas EPR20021 may target a more stable linear epitope. The observation that EPR20021 also detects FAP in a subset of tumor cells (colorectal and head & neck cancers) further supports its higher detection rate and its utility for comprehensive assessment of FAP expression. Indeed, our observation of the sensitivity and reliable performance of clone EPR20021 aligns with its wide usage in tumor microenvironment research. In recent high-resolution mapping of the tumor microenvironment, this antibody has served as a benchmark for characterizing immunosuppressive FAP+ stromal barriers [28,29], dynamic CAF subsets [30], and metabolic drug resistance mechanisms [31]. Moreover, the high reproducibility of this clone supports its use in translational applications, such as digital image analysis and targeted drug delivery systems [32,33]. Specifically, its stable staining profile has enabled standardized automated workflows using the Qupath program for computational FAP quantification in mesenchymal tumors [32], while its robust tissue-level validation guides the design of stage-specific nanoparticle therapies [33]. In addition, this tissue-level characterization provides a reliable baseline for in vivo FAPI PET/CT and SPECT/CT imaging in tumor volume definition and treatment selection [34,35]. The EPR20021 clone serves as an essential histological reference validating FAPI PET/CT target volume contouring in nasopharyngeal carcinoma radiation planning. Concurrently, ex vivo profiling with this rabbit monoclonal antibody establishes the accurate baseline required to support [^99m^Tc]Tc-FAPI SPECT/CT as a predictive biomarker for immunotherapy response in gastrointestinal cancers [34,35].

The aberrant tumor cell expression observed in head & neck and colorectal cancers adds to the growing evidence that FAP can be expressed by neoplastic cells themselves, often associated with epithelial–mesenchymal transition and poor prognosis. From a theragnostic perspective, this expands the potential target population for FAP-directed therapies. Although FAP is classically defined as a marker of stromal fibroblasts, emerging studies highlight its diverse microenvironmental localization, including expression on cancer cells across various solid tumors [1,2]. This FAP expression in tumor cells is documented in mesenchymal and neuroectodermal tumors, such as osteosarcomas, soft tissue sarcomas, and glioblastomas, particularly within the highly invasive mesenchymal subtypes [4,7]. Recent studies suggest that when FAP is expressed by tumor cells rather than only by the stroma, it may contribute to more aggressive tumor behavior [4,36,37]. Mechanistically, the presence of FAP on tumor cells has been linked to pathways involved in tissue remodeling and immune modulation [5,10]. This upregulation potentially facilitates local invasion and may play a role in drug resistance or altered treatment response [2,5]. From a clinical viewpoint, detecting FAP in both tumor cells and stroma expands the opportunities for targeted therapies [2,14]. Such dual localization potentially increases drug binding, leading to better treatment outcomes [15,27].

This study demonstrates that commercial anti-FAP clones are not interchangeable in routine pathomorphological evaluation. Although the highly significant chi-square test reflects a shared biological target, the slight diagnostic agreement (κ = 0.173) highlights a high risk of analytical misclassification. In patient screening for FAP-directed clinical trials—such as radioligand therapy selection or CAR-T cell validation—the lower detection rate of clone JA56-11 results in a 27.4% discordance rate between the two clones. Consequently, over a quarter of the patient cohort would be excluded from targeted protocols despite having verified target availability in the tumor microenvironment, as documented by the EPR20021 clone. Our results highlight differences in clone performance and support using EPR20021 for reliable, high-intensity staining.

Our study has limitations that must be acknowledged to ensure an accurate interpretation of our findings. The retrospective design and moderate sample size per cancer type, combined with the lack of clinical outcome correlation, conceptually limit the evaluation of absolute analytical sensitivity and specificity profiles, as no independent, non-immunohistochemical molecular reference standard—such as tissue microdissection followed by Western blotting or RT-qPCR—was available to confirm baseline FAP levels for each specimen. Additionally, all comparative judgments, performance characteristics, and staining trends documented herein remain restricted to the three specific commercial anti-FAP antibody clones evaluated under our standardized laboratory protocols and should not be generalized to other unexamined anti-FAP clones on the market. This consideration also applies to the 10% positivity threshold (defined as >10% vs. ≤10%), which represents an exploratory cut-off designed to assess inter-clone categorical consensus within a paired framework. Concurrently, the digital pathology validation sub-study using QuPath was restricted to a preliminary pilot cohort of ten samples, meaning that the observed spatial and quantitative correlations represent an exploratory proof-of-concept rather than a definitive confirmation of routine clinical interchangeability. Nevertheless, the clear differences between clones and the strong correlation between manual and digital scoring support the robustness of our conclusions.

## 5. Conclusions

This head-to-head comparison of three commercial anti-FAP antibodies across four common epithelial cancer types demonstrates that EPR20021 (Abcam) has higher staining intensity and detection rates in FFPE tissues, significantly outperforming JA56-11 in all four cancer types, particularly in tumors with heterogeneous or low CAF expression (breast, colorectal and head and neck cancers). Clone JA56-11 (Invitrogen) exhibits high specificity but lower staining intensity, leading to a lower detection rate in both CAFs and tumor cells compared to EPR20021 in the present experimental setting. In contrast, clone FAP/4851 showed substantial nonspecific staining under the conditions used in this study and was therefore not suitable for quantitative analysis in the present experimental setting. Importantly, aberrant tumor cell expression of FAP is a real phenomenon, detectable only by the more robustly staining EPR20021, and occurs predominantly in head and neck and colorectal cancers. The choice of antibody clone substantially shifts categorical assignment: applying an exploratory cut-off of >10% vs. ≤10% FAP-positive stromal cells, clone JA56-11 categorized as negative up to 35% of breast cancer cases that were identified as positive by clone EPR20021. These findings highlight that clones are not interchangeable under the conditions tested for standardized FAP immunohistochemistry emphasizing that future comparative designs should incorporate well-validated, highly sensitive platforms to ensure reliable and reproducible analytical stratification.

## Figures and Tables

**Table 1:** Clinical and pathological characteristics of the patient cohort.

Parameter	Breast Cancer (*n* = 29)	Lung Cancer (*n* = 42)	Head & Neck Cancer (*n* = 39)	Colorectal Cancer (*n* = 27)
Age, years (mean ± SD)	58.9 ± 9.7	63.8 ± 8.2	61.2 ± 11.0	63.2 ± 9.9
Sex, n (%)				
Male	0 (0)	37 (88)	32 (82)	13 (48)
Female	29 (100)	5 (12)	7 (18)	14 (52)
pT stage, n (%)				
pT1	8 (28)	11 (26)	14 (36)	4 (15)
pT2	13 (45)	12 (29)	12 (31)	7 (26)
pT3	5 (17)	13 (31)	9 (23)	9 (33)
pT4	3 (10)	6 (14)	4 (10)	7 (26)
pN stage, n (%)				
pN0	12 (41)	10 (24)	25 (64)	6 (22)
pN1	10 (35)	15 (36)	8 (21)	11 (41)
pN2	5 (17)	11 (26)	5 (13)	7 (26)
pN3	2 (7)	6 (14)	1 (3)	3 (11)
Lymph node metastases, n (%)	18 (62)	32 (76)	14 (36)	21 (78)
Distant metastases, n (%)	3 (10)	5 (12)	0 (0)	0 (0)
Neoadjuvant therapy, n (%)	6 (21)	2 (5)	2 (5)	9 (33)
Recurrence, n (%)	7 (24)	4 (10)	6 (15)	4 (15)

**Table 2:** Detailed characteristics of the anti-FAP antibodies used in the study.

Parameter	Antibody 1	Antibody 2	Antibody 3
Clone	FAP/4851	JA56 11	EPR20021
Host/Isotype	Mouse/IgG2c, kappa	Rabbit/IgG	Rabbit/IgG
Immunogen	Recombinant fragment (aa 1–200) of human FAP	Recombinant protein (aa 1–160) of human FAP	Not disclosed (Recognizes 95 kDa and glycosylated 100–120 kDa forms)
Species reactivity	Human	Human	Human
Clonality	Monoclonal	Monoclonal	Monoclonal
Manufacturer (Cat. No.)	Invitrogen (2191-MSM1-P0; Carlsbad, CA, USA)	Invitrogen (MA5-32670; Carlsbad, CA, USA)	Abcam (ab207178; Cambridge, UK)
Optimal dilution (IHC)	1:1000	1:800	1:300
Antigen retrieval	Tris-EDTA	Tris-EDTA	Tris-EDTA
Expected MW (Western blot)	~95 kDa	~95 kDa	95 kDa + 100–120 kDa (glycosylated)

**Table 3:** Comparative analysis of FAP expression in the stromal across different cancer types.

Cancer Type	Antibody	Median H-Score (IQR)	*p*-Value (H-Score)	Median % Positive Cells (IQR)	*p*-Value (% Positive)
Colorectal (*n* = 27)	EPR20021	60 (10–160)	**0.0001**	50 (10–75)	**0.0010**
	JA56-11	10 (1–50)		10 (1–30)	
Lung (*n* = 42)	EPR20021	145 (50–180)	**0.0002**	77.5 (50–90)	**0.0004**
	JA56-11	55 (0–112.5)		50 (0–76.25)	
Breast (*n* = 29)	EPR20021	100 (50–200)	**<0.0001**	80 (50–100)	**<0.0001**
	JA56-11	10 (0–40)		10 (0–40)	
Head & Neck (*n* = 39)	EPR20021	80 (50–160)	**0.0179**	75 (50–90)	0.0539
	JA56-11	50 (30–100)		50 (30–75)	

Data for FAP/4851 are excluded from quantitative analysis due to nonspecific binding and are presented for qualitative comparison only (Section 3.1 and Figure 1). *p*-values refer to the pairwise comparison between EPR20021 and JA56-11 using the Wilcoxon signed-rank test. Statistically significant values (*p* < 0.05) are highlighted in bold. IQR = interquartile range (Q1–Q3).

**Table 4:** Comparative analysis of FAP expression in the tumor cell compartment across different cancer types.

Cancer Type	Antibody	FAP-Positive Cases, n (%)	Median H-Score (IQR)	*p*-Value (H-Score)	Median % Positive Cells (IQR)	*p*-Value (% Positive)
Colorectal (*n* = 27)	EPR20021 JA56-11	16 (59%) 0 (0%)	0 (0–10) 0 (0–0)	**0.0015**	0 (0–10) 0 (0–0)	**0.0015**
Lung (*n* = 42)	EPR20021 JA56-11	14 (33%) 1 (2.4%)	0 (0–1.25) 0 (0–0)	0.3492	0 (0–1.25) 0 (0–0)	0.3804
Breast (*n* = 29)	EPR20021 JA56-11	1 (3%) 0 (0%)	0 (0–0) 0 (0–0)	0.2500	0 (0–0) 0 (0–0)	0.2500
Head & Neck (*n* = 39)	EPR20021 JA56-11	23 (59%) 2 (5.1%)	5 (0–25) 0 (0–0)	0.1955	5(0–20) 0 (0–0)	0.3274

*p*-values refer to the pairwise comparison between EPR20021 and JA56-11 using the Wilcoxon signed-rank test. Statistically significant values (*p* < 0.05) are highlighted in bold. IQR = interquartile range (Q1–Q3).

## Data Availability

The raw data of this article will be made available by the authors upon reasonable request, without undue reservation.

## Supplementary Materials

The following supporting information can be downloaded at https://www.mdpi.com/article/10.3390/antib15050093/s1, Figure S1: Immunohistochemical specificity profiling of anti-FAP antibody clones in positive and negative controls.

## Abbreviations

The following abbreviations are used in this manuscript:

CAF	Cancer-associated fibroblast
CI	Confidence interval
ECM	Extracellular matrix
FAP	Fibroblast activation protein
FFPE	Formalin-fixed paraffin-embedded
H&N	Head and neck
IHC	Immunohistochemistry
IQR	Interquartile range
LoA	Limits of agreement
RLT	Radioligand therapy
TME	Tumor microenvironment
