# Supplementary material for: Head-to-Head Comparison of FAP Antibody Clones for Tissue Expression Analysis
Source: Antibodies (Basel). 2026 Oct 8;15(5):93. doi: 10.3390/antib15050093 (PMC13700842; doi:10.3390/antib15050093)
Supplement: Supplementary file 1 [file antibodies-15-00093-s001.zip › antibodies-4532501-supplementary.pdf]

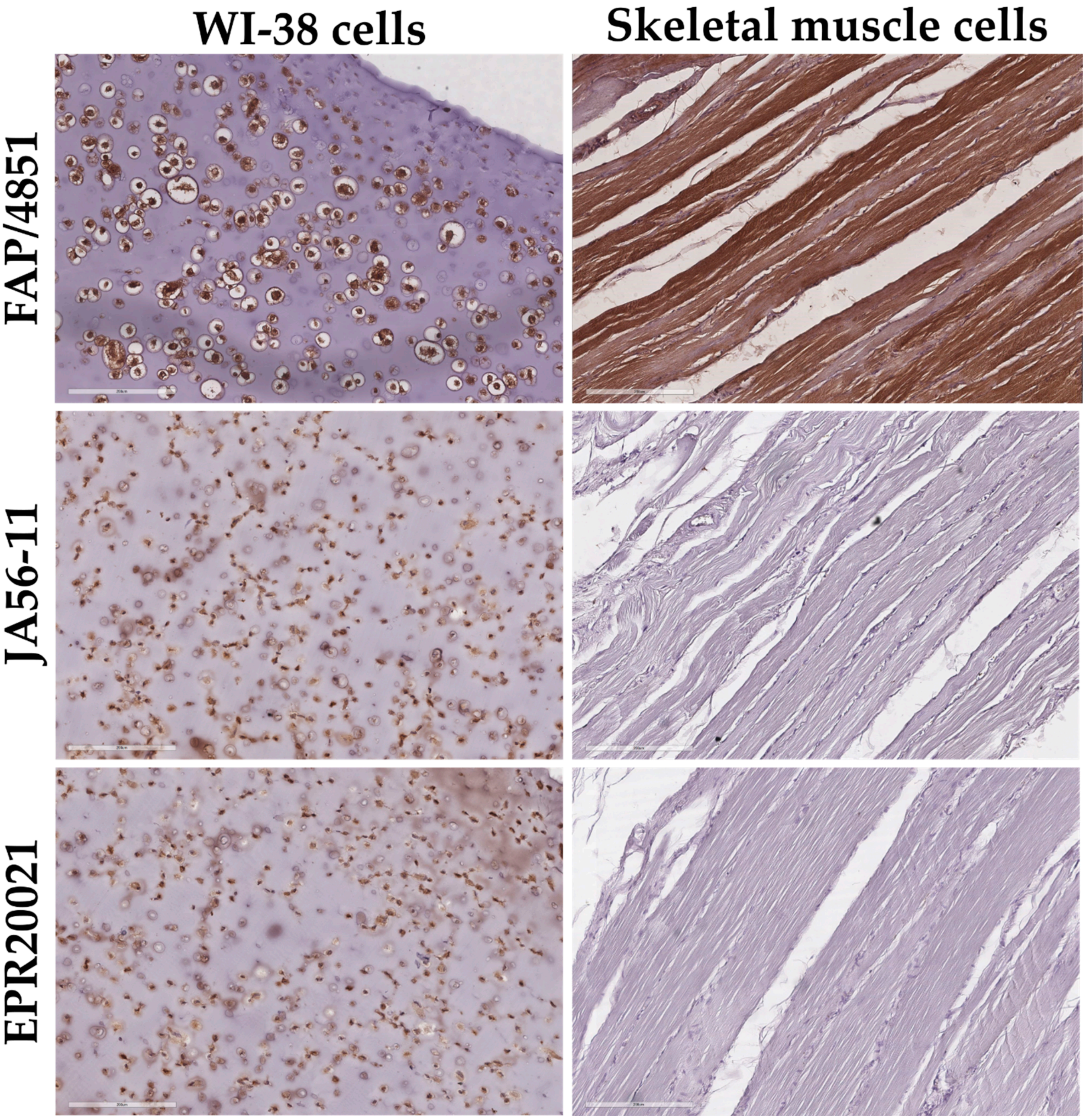

Figure S1. Immunohistochemical specificity profiling of anti-FAP antibody clones in positive and negative controls. Micrographs show the staining patterns of the antibody clones (rows) across control platforms (columns). Columns display normal human skeletal muscle (negative control) and a gelatin-based cell block of WI-38 human fibroblasts (positive control). Clones EPR20021 and JA56-11 demonstrate high specificity, showing strong membranous and cytoplasmic staining in WI-38 cells and a total absence of immunoreactivity in skeletal muscle. In contrast, the non-specific clone FAP/4851 exhibits false-positive reactivity within the negative control muscle tissue. Baseline non-specific noise is verified via an isotype-matched negative control. Scale bars represent 200 μm.
